# Adult Human Multipotent Neural Cells Could Be Distinguished from Other Cell Types by Proangiogenic Paracrine Effects via MCP-1 and GRO

**DOI:** 10.1155/2021/6737288

**Published:** 2021-08-12

**Authors:** Sung Soo Kim, Hee-Jang Pyeon, Yoon Kyung Bae, Hyun Nam, Chung Kwon Kim, Sun-Ho Lee, Kyeung Min Joo

**Affiliations:** ^1^Department of Health Sciences and Technology, Samsung Advanced Institute for Health Sciences & Technology (SAIHST), Samsung Medical Center, Sungkyunkwan University School of Medicine, Seoul 06351, Republic of Korea; ^2^Stem Cell and Regenerative Medicine Institute, Research Institute for Future Medicine, Samsung Medical Center, Sungkyunkwan University School of Medicine, Seoul 06351, Republic of Korea; ^3^Single Cell Network Research Center, Sungkyunkwan University School of Medicine, Suwon 16419, Republic of Korea; ^4^Department of Anatomy & Cell Biology, Sungkyunkwan University School of Medicine, Suwon 16419, Republic of Korea; ^5^Medinno Research Institute, Medinno Inc., Suwon 16419, Republic of Korea; ^6^Department of Neurosurgery, Samsung Medical Center, Sungkyunkwan University School of Medicine, Seoul 06351, Republic of Korea; ^7^Biomedical Institute for Convergence at Sungkyunkwan University (BICS), Sungkyunkwan University, Suwon 16419, Republic of Korea

## Abstract

Adult human multipotent neural cells (ahMNCs) are unique cells derived from adult human temporal lobes. They show multipotent differentiation potentials into neurons and astrocytes. In addition, they possess proangiogenic capacities. The objective of this study was to characterize ahMNCs in terms of expression of cell type-specific markers, *in vitro* differentiation potentials, and paracrine factors compared with several other cell types including fetal neural stem cells (fNSCs) to provide detailed molecular and functional features of ahMNCs. Interestingly, the expression of cell type-specific markers of ahMNCs could not be differentiated from those of pericytes, mesenchymal stem cells (MSCs), or fNSCs. In contrast, differentiation potentials of ahMNCs and fNSCs into neural cells were higher than those of other cell types. Compared with MSCs, ahMNCs showed lower differentiation capacities into osteogenic and adipogenic cells. Moreover, ahMNCs uniquely expressed higher levels of MCP-1 and GRO family paracrine factors than fNSCs and MSCs. These high levels of MCP-1 and GRO family mediated *in vivo* proangiogenic effects of ahMNCs. These results indicate that ahMNCs have their own distinct characteristics that could distinguish ahMNCs from other cell types. Characteristics of ahMNCs could be utilized further in the preclinical and clinical development of ahMNCs for regenerative medicine. They could also be used as experimental references for other cell types including fNSCs.

## 1. Introduction

Neural stem cell (NSC) is a promising regenerative modality for various neurodegenerative and neurological disorders based on its differentiation potentials into functional neural cells [1–6]. Several reliable sources of human NSCs including embryonic stem cells (ESCs) [7, 8], induced pluripotent stem cells (iPSCs) [9, 10], fetal brains [11–14], and adult brains [15–17] have been introduced for NSC therapy. NSCs could also be directly converted from fully differentiated cells by genetic modifications [18–20]. However, differences in characteristics of NSCs from different sources need to be elucidated further to understand therapeutic effects and treatment mechanisms of NSCs.

Previously, we have successfully isolated and cultured adult human multipotent neural cells (ahMNCs) from adult human temporal lobes [15, 21, 22]. They can be differentiated into neurons and astrocytes. They also showed significant therapeutic effects in animal models of ischemic stroke [15] and spinal cord injury (SCI) [22]. Interestingly, in damaged spinal cords, transplanted ahMNCs had significant proangiogenic effects, which might be one of the therapeutic mechanisms of ahMNCs for SCI [22]. Since proangiogenic effects might not be expected for NSCs, ahMNCs might have unique paracrine mediators that could have functional effects on the microenvironment of the central nervous system (CNS).

Several studies have reported that pericytes in adult human brains might be the source of NSCs that can proliferate upon damage in the CNS [23–29]. Moreover, pericytes have strong proangiogenic activities [30–32] that are also observed in mesenchymal stem cells (MSCs) [33–35]. Given their similar paracrine effects, we hypothesized that ahMNCs might have features that are shared with pericytes and/or MSCs. Accordingly, the expression of surface markers, *in vitro* differentiation potentials, and paracrine factors of ahMNCs were compared with those of fetal NSCs (fNSCs), pericytes, and MSCs in this study. Results indicated that ahMNCs had distinct features in terms of proangiogenic factors and differentiation potentials, although ahMNCs also shared some common surface markers with fNSCs, pericytes, and MSCs. Here, we elucidated that ahMNCs could exert proangiogenic effects via excretion of MCP-1 and GRO.

## 2. Materials and Methods

### 2.1. Study Approval

To acquire surgical samples for stem cell culture, informed written consent was obtained from each patient according to guidelines approved by the Institutional Review Board of Samsung Medical Center (SMC, Seoul, Korea) (IRB file numbers: SMC 2009-07-071-027, 2016-09-120, and 2015-03-061). Animal experiments were approved by the Institutional Animal Care and Use Committee of Samsung Biomedical Research Institute (Seoul, South Korea) (approval number: 20140916001) and were conducted in accordance with the “National Institute of Health Guide for the Care and Use of Laboratory Animals” (eighth edition, revised in 2011).

### 2.2. Cell Isolation and Culture

We followed the methods of Lee et al. [22]; fNSCs were purchased from Millipore (Billerica, MA, USA) and maintained in ReNcell maintenance medium (Millipore) supplemented with 20 ng/ml human epidermal growth factor (EGF) (R&D Systems; Minneapolis, MN, USA), 20 ng/ml human basic fibroblast growth factor (bFGF) (R&D Systems), and 100 U/ml penicillin/streptomycin (P/S) (Corning Inc.; NY, USA). Human pericytes from placenta (hPC-PL) and human umbilical vein endothelial cells (HUVECs) were purchased from PromoCell (Heidelberg, Germany) and cultured in pericyte growth medium (PGM) (PromoCell) and endothelial growth medium (EGM) (PromoCell), respectively. Human dental pulp-derived mesenchymal stem cells (DPSCs) were obtained from Prof. Lee of Dental Research Institute, Seoul National University (Seoul, South Korea). Culture of DPSCs was conducted as described previously [36]. Human adipose tissue-derived mesenchymal stem cells (ADSCs) were obtained from Prof. Oh of Adipose Stem Cell Bank, Seoul Mary's Hospital of Korea, and cultured in Dulbecco's low-glucose modified Eagle's medium (DMEM/low glucose) (Hyclone; Road Logan, UT, USA) supplemented with 10% FBS (Gibco; Paisley, Scotland, UK) and 100 U/ml P/S.

### 2.3. Collection of Conditioned Medium

After 2 × 10^5^ ahMNCs, fNSC, DPSCs, or ADSCs at in vitro passage 6 were plated into 55 cm^2^ culture dishes and incubated for 2 days, cells were maintained in basal medium: Dulbecco's modified Eagle's medium : Nutrient Mixture F-12 (DMEM/F12) (Gibco) for ahMNCs, ReNcell maintenance medium for fNSCs, Minimum Essential Medium Alpha Modification (*α*-MEM) (Hyclone) for DPSCs, and DMEM/low glucose for ADSCs. After culturing for 24 h, the conditioned medium (CM) was collected.

### 2.4. In Vitro Tube Formation Assay and In Vivo Matrigel Plug Assay

In vitro tube formation assay was performed using growth factor-reduced and phenol red-free Matrigel (BD Biosciences; Franklin Lakes, NJ, USA) according to the manufacturer's instructions. Briefly, 2.5 × 10^4^ HUVECs in EGM were seeded onto Matrigel-coated (50 *μ*l/well) 96-well plates. After 12 h of incubation, the culture medium was fully replaced with CM of ahMNCs (from 0% to 100%, diluted with DMEM/F12). At 18 h after CM treatment, total tube length and mesh area per well were measured using ImageJ software (National Institute of Health; MD, USA, https://imagej.nih.gov/ij/index.html). For in vivo Matrigel plug assay, 2 × 10^6^ cells at ratios of 100 : 0, 50 : 50, or 0 : 100 (HUVECs : ahMNCs) were suspended in 200 *μ*l of ice-cold phenol red-free Matrigel (BD Bioscience). Matrigel that was in semiliquid solution at 4°C solidified after in vivo transplantation, which could affect the morphologies of Matrigel. To prepare green fluorescent protein- (GFP-) expressing cells, a lentiviral system (Life Technologies; Carlsbad, CA, USA) was utilized according to previous studies [37, 38]. These cell-Matrigel mixtures were transplanted subcutaneously into the dorsal surface of immune-deficient Balb-c/nu mice (6-week-old, male) (Orient Bio; Seongnam, South Korea) under isoflurane (Ifran™, Hana Pharm, Seoul, South Korea) anesthesia. Full anesthesia was confirmed every 5 minutes by loss of the righting reflex and no response to painful stimuli on a back paw. Possible toxic effects of the analgesics were monitored by observation of respiratory movement [39]. No toxic effects of anesthesia were observed in this study. During experimental procedures, the mice were placed on a heating pad that was preheated to 37°C. The animals were housed under a 12 h/12 h light/dark cycle and were provided with pellet food and distilled water. At 4 days after injection, Matrigel plugs were removed, fixed with 10% buffered formalin, and embedded in paraffin. Paraffin blocks were sectioned (4-*μ*m thick) using Leica RM 2165 microtome (BIORP, Leica, Nussloch, Germany). Antigens were retrieved at boiling temperature for 30 min in antigen retrieval solution (Dako; Glostrup, Denmark). Sections were treated with primary antibodies (Table SI) overnight at 4°C. Nuclei were stained with DAPI (Invitrogen) for 5 min at room temperature (RT). To block activities of MCP-1 and GRO*α*/*β*/*γ* in in vivo Matrigel plug assay, 1 *μ*g/ml, 5 *μ*g/ml, or 10 *μ*g/ml of neutralizing antibodies for MCP-1 and GRO*α*/*β*/*γ* (R&D Systems) were added to the Matrigel.

### 2.5. Quantitative Real-Time PCR (qRT-PCR)

Total RNA was extracted using RNeasy Mini Kits (Qiagen, Valencia, CA, USA). cDNA was generated by reverse transcription of total RNA (5 *μ*g) using Tetro cDNA Synthesis Kit (BIOLINE; London, UK) following the manufacturer's protocol. Reaction mixture (15 *μ*l) for qRT-PCR included 100 ng cDNA, 0.33 pM primers (Table SII), and 5× HOT FIREPol EvaGreen qPCR Mix Plus (ROX) (Solis BioDyne; Tartu, Estonia). GAPDH was used for qRT-PCR normalization.

### 2.6. Flow Cytometric Analysis

Cells were suspended in Dulbecco's phosphate-buffered saline (DPBS) (Welgene; Dae-gu, South Korea) containing 2% FBS and fixed with BD Cytofix Fixation Buffer for 20 min at RT. Antibodies (Table SIII) were applied for 30 min on ice.

### 2.7. In Vitro Differentiation

Osteogenic differentiation medium was *α*-MEM supplemented with 5% FBS, 50 *μ*g/ml L-ascorbic acid phosphate, 10 mM *β*-glycerophosphate, and 0.1 *μ*M dexamethasone (Sigma-Aldrich). Adipogenic differentiation was induced with StemPro Adipogenesis Differentiation Kit (Gibco). Cells were maintained in the differentiation medium for 17 days. As a control, cells were cultured in *α*-MEM supplemented with 5% FBS. Calcium deposit and lipid vacuoles were stained with Alizarin red S and Oil red O solution (Sigma-Aldrich), respectively. For neural differentiation, cells at 70~80% confluency were cultured in DMEM/F12 supplemented with 100 U/ml P/S, 1× B-27 Supplement (Gibco), and 0.5 mM 3-isobutyl-1-methylxanthine (IBMX) (Gibco) for two days. For immunocytochemistry (ICC), cells were fixed with ice-cold 4% paraformaldehyde (Biosesang; Gyeonggi, South Korea) for 20 min at RT. Cells were then incubated with primary antibodies (Table SI) overnight at 4°C.

### 2.8. Human Cytokine Array and ELISA

CMs were analyzed with a semiquantitative human cytokine array (RayBio Human Cytokine Antibody Array C Series 1000, RayBiotech; Norcross, GA, USA) according to the manufacturer's instructions. To verify results, CMs were also assayed with enzyme-linked immunosorbent assay (ELISA) for MCP-1 and GRO*α* using Quantikine ELISA Kits (R&D Systems) according to the manufacturer's instructions.

### 2.9. Statistics

Data were analyzed using statistical software R, version 3.6.0 (The R Foundation; Vienna, Austria, https://www.r-project.org/). Data are presented as average ± standard deviation (SD). Differences were analyzed by two-tailed Student's *t*-test. Statistical significance was considered at *P* < 0.05. For multiple comparisons, *P* values from two-tailed Student's *t*-test were adjusted by a post hoc test Bonferroni correction.

## 3. Results

### 3.1. In Vitro and In Vivo Proangiogenic Paracrine Effects of ahMNCs

**Figure 1:**
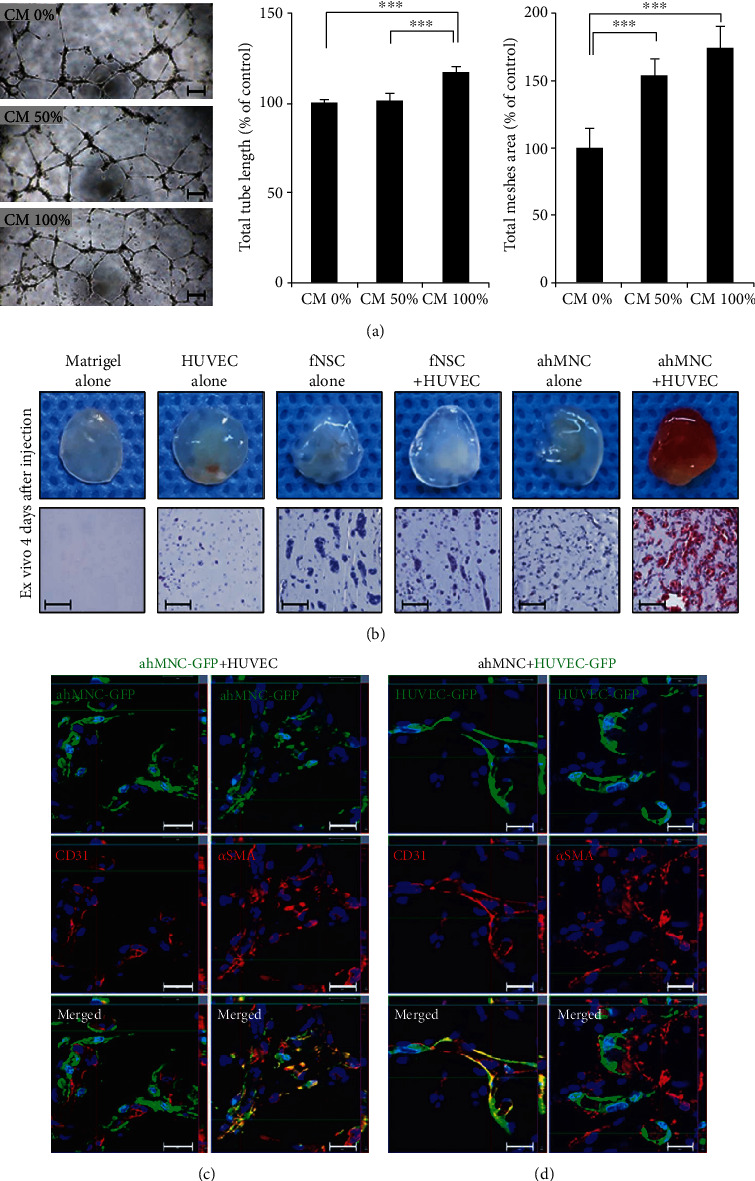
Proangiogenic activities of ahMNCs. (a) *In vitro* tube formation assay. Different ratios of CM of ahMNCs were applied to HUVECs in Matrigel for 18 h. Tube length and mesh area were analyzed and compared. Scale bar, 200 *μ*m. Height = average, error bar = SD. ^∗∗∗^*P* < 0.01. Representative data (001TL) of triplicated experiments are shown. (b) *In vivo* Matrigel plug assay. 2 × 10^6^ cells at ratios of 100 : 0, 50 : 50, or 0 : 100 (HUVECs : fNSCs or HUVEC : ahMNCs) in Matrigel were transplanted subcutaneously into dorsal surfaces of Balb-c/nu mice. Four days after transplantation, *in vivo* angiogenesis was analyzed histologically. Scale bar, 100 *μ*m. Representative data (015TL) of triplicated experiments are illustrated. (c, d) After transplantation of ahMNCs tagged with GFP and HUVEC (c), or ahMNCs and HUVEC tagged with GFP (d), their localization was analyzed by immunofluorescence. Scale bars, 20 *μ*m. Representative data (001TL) of triplicated experiments are illustrated.

Three batches of ahMNCs (001TL, 008TL, and 015TL) were isolated from different human temporal lobes that were removed surgically to control localized temporal lobe epilepsy (cortical dysplasia type IIIa). These ahMNCs were then primarily cultured as previously reported [15, 21, 22]. To test *in vitro* proangiogenic paracrine effects of ahMNCs, conditioned medium (CM) from ahMNCs was applied to HUVEC human endothelial cells (Figure 1(a)). Compared to the control medium, CM increased both tube length and meshed area of HUVECs significantly, which also showed a dose dependency (Figure 1(a)). In *in vivo* Matrigel plug assay, ahMNCs showed dramatic proangiogenic effects when ahMNCs were cotransplanted with HUVECs (Figure 1(b)). In contrast, *in vivo* vessel formation was not observed when fNSCs were injected with HUVECs (Figure 1(b)). Moreover, HUVEC, ahMNC, or fNSC alone group had little angiogenic activities *in vivo* (Figure 1(b)). These results confirmed that ahMNCs possessed unique proangiogenic paracrine effects.

When *in vivo* fate of ahMNCs was traced in *in vivo* Matrigel plug assay, GFP expressed by ahMNCs was colocalized with alpha-smooth muscle actin (*α*SMA), a specific marker of pericytes, but not with CD31, a specific marker of endothelial cells (Figure 1(c)). In contrast, GFP expressed by HUVECs was colocalized with CD31, but not with *α*SMA (Figure 1(d)). These results indicated that ahMNCs cotransplanted with HUVECs might have similar characteristics with those of pericytes. In addition, unique proangiogenic paracrine effects of ahMNCs were not mediated by *trans*-differentiation of ahMNCs into endothelial cells.

### 3.2. Lineage-Specific Marker Expression of ahMNCs

**Figure 2:**
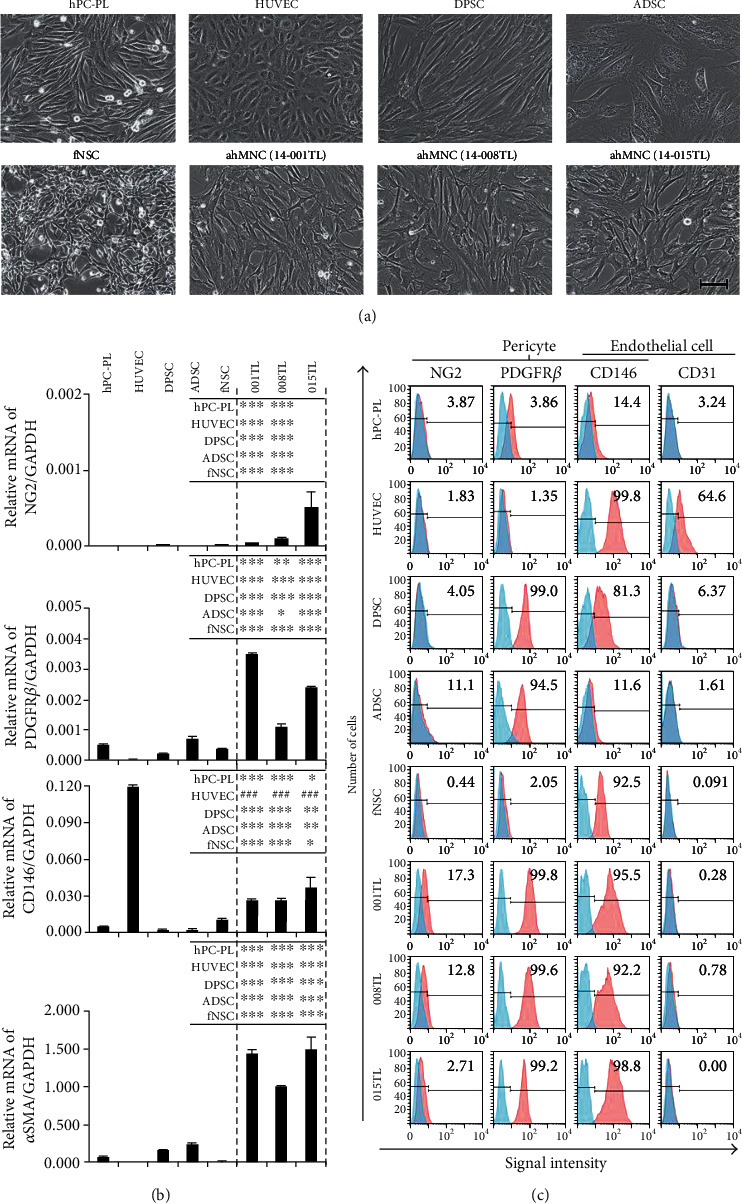
Expression of pericyte markers in ahMNCs. (a) Morphologies of cell types tested were illustrated. Scale bar, 100 *μ*m. (b) Levels of mRNAs of pericyte markers were analyzed by RT-qPCR. Height = average, error bar = SD. Inlet tables indicate statistical significance between ahMNCs and other cell types. ^∗^ and ^#^ represent significantly higher and lower levels in ahMNCs, respectively. ^∗^*P* < 0.05, ^∗∗^*P* < 0.03, ^∗∗∗^*P* < 0.01, and ^###^*P* < 0.01. (c) Protein levels of NSC (NG2), pericyte (NG2, PDGFR*β*, and CD146) or endothelial cell (CD146 and CD31) markers were analyzed by flow cytometry. Number in each panel indicated percent of marker-positive cells.

Since ahMNCs were localized at the position of pericytes and expressed *α*SMA in the Matrigel plug assay, we hypothesized that ahMNCs might have features of pericytes or mesenchymal cells. To elucidate characteristics of ahMNCs, cell morphology and lineage-specific markers of ahMNCs were compared with those of hPC-PL human placenta-derived pericytes, HUVECs, MSCs (DPSCs and ADSCs), and fNSCs. hPC-PL and HUVEC were used as representative pericytes and endothelial cells, respectively, since they were commercially available and relatively well characterized. Morphologically, shapes of ahMNCs were more diverse and cell sizes of ahMNCs were bigger than those of fNSCs (Figure 2(a)).

At first, mRNA levels of pericyte markers such as neuron-glial antigen 2 (NG2), platelet-derived growth factor receptor-beta (PDGFR*β*), CD146, and *α*SMA were analyzed. Three batches of ahMNCs showed significantly higher levels of PDGFR*β*, CD146, and *α*SMA mRNAs compared to other cell types (Figure 2(b)), whereas CD146 mRNA level of HUVECs was significantly higher than those of ahMNCs. Levels of NG2 mRNA were relatively low in ahMNCs; however, those of 001TL and 008TL were significantly higher than those of other cell types (Figure 2(b)). In contrast, mRNA levels of pericyte markers in fNSCs and MSCs were comparable with those in hPC-PL (Figure 2(b)). When protein levels were analyzed by flow cytometry (Figure 2(c)), expression patterns of pericyte markers at protein level were similar to those at mRNA level (Figure 2(b)). Notably, NG2 expression at mRNA and protein levels was only detected in ahMNCs (Figures 2(b) and 2(c)), whereas expression of CD31, an endothelial cell-specific marker, was only observed in HUVECs (Figure 2(c)). Between the three batches of ahMNCs, 008TL showed significantly higher NG2 mRNA and significantly lower PDGFR*β* and *α*SMA mRNA than those of 001TL (Figure 2(b)). However, at protein level, the differences were not observed (Figure 2(c)).

**Figure 3:**
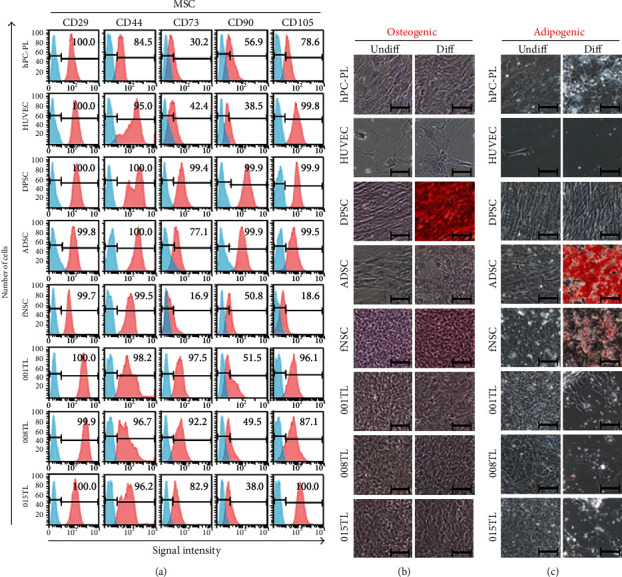
Expression of MSC markers and *in vitro* differential potential into mesenchymal lineage cells of ahMNCs. (a) Protein levels of MSC markers were analyzed by flow cytometry. Number in each panel indicated percent of marker-positive cells. (b, c) Cells were maintained in osteogenic (b) or adipogenic (c) differentiation medium for 17 days (Diff). Undifferentiated cells (Undiff) were maintained in *α*-MEM supplemented with 5% FBS for 17 days. (b, c) Calcium deposit (b) and lipid vacuoles (c) were then stained with Alizarin red S and Oil red O solution, respectively. Scale bars, 100 *μ*m.

When the expression of MSC markers (CD29, CD44, CD73, CD90, and CD105) and hematopoietic stem cell (HSC) markers (CD14, CD45, and CD117) was analyzed by flow cytometry, all cell types showed high levels of MSC markers (Figure 3(a)) but low levels of HSC markers (Fig. S1). Validity of flow cytometric analysis results was confirmed using positive control (HLA-I) and negative control (HLA-DR) (Fig. S1). Taken together, these results suggest that ahMNCs have pericyte- and MSC-like characteristics in the expression of cell type-specific markers.

### 3.3. In Vitro Differentiation Potentials of ahMNCs

Since ahMNCs expressed high levels of pericyte and MSC markers, we further analyzed *in vitro* differentiation potentials of ahMNCs into osteogenic- and adipogenic-mesenchymal lineage cells. Under differentiation conditions, DPSCs and ADSCs made abundant calcium deposits (Figure 3(b)) and lipid droplets (Figure 3(c)), respectively. Compared with DPSCs, ADSCs showed higher adipogenic potential (Figure 3(b)) but lower osteogenic capability (Figure 3(c)). In contrast, few HUVECs were alive under differentiation conditions (Figures 3(b) and 3(c)). hPC-PL and fNSCs had low differentiation potentials into osteogenic-mesenchymal (Figure 3(b)) and adipogenic-mesenchymal (Figure 3(c)) mesenchymal lineage cells. Three batches of ahMNCs also showed little *in vitro* differentiation potentials into osteogenic (Figure 3(b)) and adipogenic (Figure 3(c)) cells. Those results from 3-repeated experiment indicate that ahMNCs have MSC-like properties only in terms of cell type marker expression.

**Figure 4:**
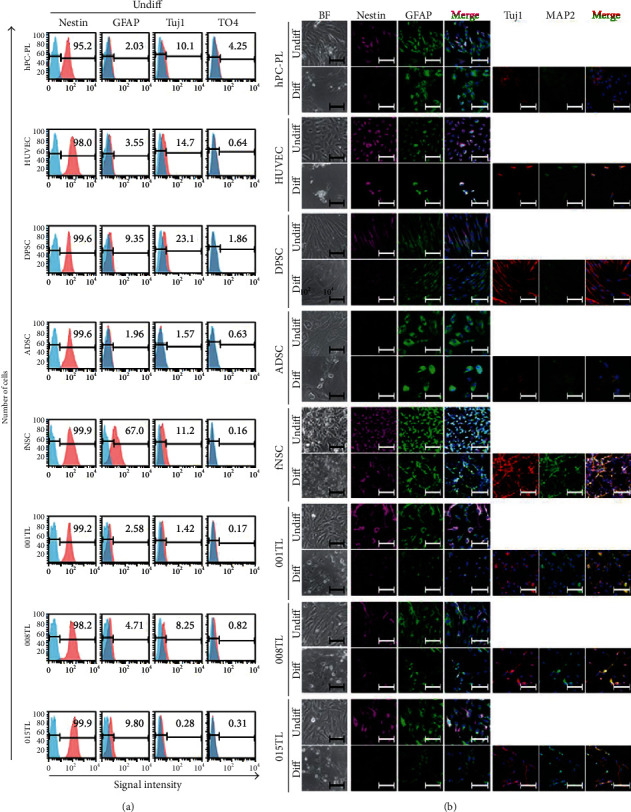
Expression of NSC-related markers and *in vitro* differential potential into neural cells of ahMNCs. (a) Protein levels of NSC (Nestin) and neural cell (Tuj1 for neurons, O4 for oligodendrocytes, and GFAP for astrocytes) markers were analyzed by flow cytometry. Number in each panel indicated percent of marker-positive cells. (b) Expression of NSC (Nestin) and neural cell (Tuj1 and MAP2 for neurons and GFAP for astrocytes) markers of cells before (Undiff) or after neural differentiation for 2 days (Diff) were analyzed by immunocytochemistry. BF: bright field. Scale bars, 100 *μ*m.

To confirm the neurogenic potential of ahMNCs compared to those of other cell types, expression levels of NSC markers and *in vitro* differentiation potentials into neural cells were analyzed by flow cytometry and immunocytochemistry (ICC), respectively. Under undifferentiated condition, Nestin, an NSC marker, was expressed in all cell types analyzed, whereas the level of glial fibrillary acidic protein (GFAP), an astrocyte marker, was higher in fNSCs than that in other cell types (Figure 4(a)). In ICC, Nestin was not observed in ADSCs while GFAP was detected in all cell types, although the expression level of GFAP was the highest in fNSCs (Figure 4(b)). Neuron-specific class III beta-tubulin (Tuj1), a neuron marker, and O4, an oligodendrocyte marker, showed low expression levels in all cell types (Figure 4(a)). Under differentiation condition for neural cells, only a few HUVECs and ADSCs survived (Figure 4(b)). Expression level of Nestin in all cell types decreased after differentiation (Figure 4(b)). Tuj1 and microtubule-associated protein 2 (MAP2) as neuron markers were detected in all cell types after differentiation (Figure 4(b)). However, levels of neuron markers in fNSCs and ahMNCs were much higher than those in other cell types. Moreover, only fNSCs and ahMNCs showed differentiated neural cell morphologies (Figure 4(b)). Expression of GFAP, an astrocyte marker, was not changed much after differentiation in any cell type (Figure 4(b)). After differentiation, 80–90% of ahMNCs were GFAP-positive and 10–20% of cells were Tuj1 and/or MAP2-positive (Figure 4(b)), which was similar with the previous report [15]. These results indicate that *in vitro* neural cell differentiation potentials of fNSCs and ahMNCs are higher than those of hPC-PL, HUVECs, and MSCs.

### 3.4. Paracrine Mediators of ahMNCs

**Figure 5:**
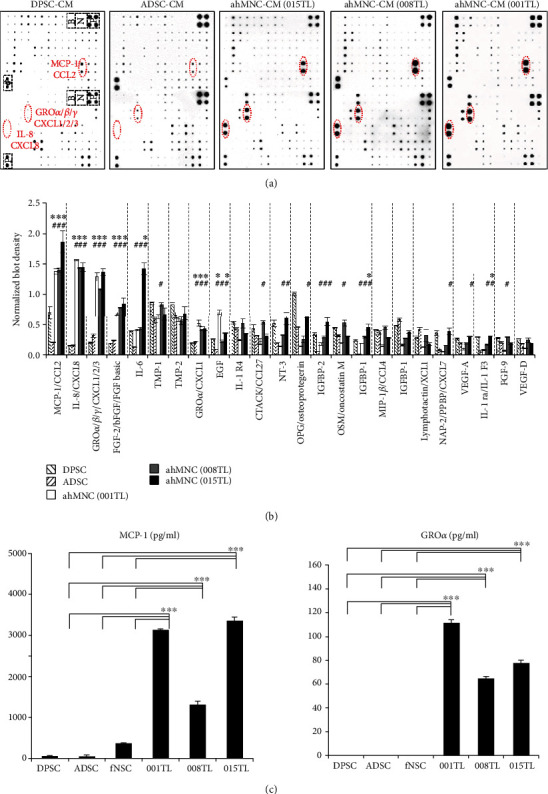
Paracrine factors of ahMNCs. (a) 120 paracrine factors of ahMNCs, DPSCs, and ADSCs were analyzed by cytokine array. (b) Cytokines whose levels were within top 20% in the CM of ahMNCs were illustrated and compared with those of DPSCs and ADSCs. Height = average, error bar = SD. ^∗^ and ^#^ indicate significantly higher level of cytokine in ahMNCs compared to DPSCs and ADSCs, respectively. ^∗^*P* < 0.01 and ^#^*P* < 0.05. (c) Protein levels of MCP-1 and GRO*α* in CM were analyzed by ELISA and then compared. Height = average, error bar = SD. ^∗∗∗^*P* < 0.01.

To elucidate mechanisms involved in the proangiogenic effects of ahMNCs, 120 paracrine mediators of ahMNCs were further compared with those of MSCs (DPSCs and ADSCs) by cytokine array (Figure 5(a)). Among these cytokines, MCP-1 and GRO family have been reported as potent proangiogenic factors [40–50], indicating that MCP-1 and GRO family might mediate the proangiogenic effects of ahMNCs. Interestingly, MCP-1 and GRO family in the CM of 001TL, 008TL, and 015TL were significantly higher than those in the CM of DPSCs and ADSCs (Figure 5(b)), which suggested that molecular mechanisms of proangiogenic effects of ahMNCs might be different from those of DPSCs and ADSCs. Differences in cytokine levels among the three batches of ahMNCs were summarized in Table SIV. Specific high excretion of MCP-1 and GRO*α* of ahMNCs was confirmed by ELISA compared with DPSCs, ADSCs, and fNSCs (Figure 5(c)). Taken together, these results suggest that ahMNCs can release unique paracrine proangiogenic mediators.

### 3.5. Functions of MCP-1 and GRO Family in Paracrine Proangiogenic Activities of ahMNCs

**Figure 6:**
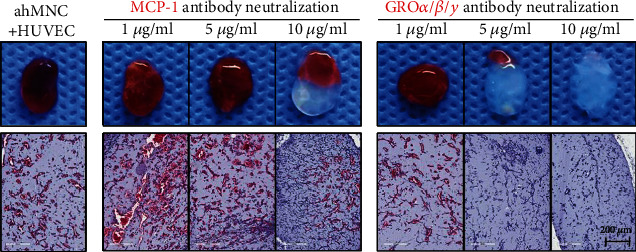
Paracrine mediators of *in vivo* proangiogenic activities of ahMNCs. 1 × 10^6^ HUVECs and 1 × 10^6^ ahMNCs in Matrigel were transplanted subcutaneously into dorsal surfaces of Balb-c/nu mice, to which 0, 1, 5, or 10 *μ*g/ml MCP-1 or GRO*α*/*β*/*γ* neutralizing antibody was applied. Four days after transplantation, *in vivo* angiogenesis was analyzed histologically. Representative data (001TL) of triplicated experiments are shown.

To test the hypothesis that MCP-1 and GRO family might mediate specific proangiogenic effects of ahMNCs, *in vivo* Matrigel plug assay was performed with or without anti-MCP-1 and/or GRO family neutralizing antibodies. While cotransplantation of ahMNCs and HUVECs induced robust *in vivo* micro-vessel formation, anti-MCP-1 or GRO family neutralizing antibody significantly and dose dependently inhibited the proangiogenic effects of ahMNCs (Figure 6). Taken together, these results suggest that specific proangiogenic activities of ahMNCs are mediated by MCP-1 and GRO family.

## 4. Discussion

In this study, we characterized ahMNCs in detail in terms of expression of cell type-specific markers, *in vitro* differentiation potentials into mesenchymal or neuroepithelial lineage cells, and excretion of paracrine factors, compared with pericytes, endothelial cells, MSCs, and fNSCs. Expression pattern of cell type-specific markers of ahMNCs showed that ahMNCs have pericyte- and MSC-like characteristics. Expression levels of pericyte makers in ahMNCs were even higher than those in pericytes. However, ahMNCs also showed its distinct features such as cellular morphology and NG2 expression compared to MSCs and pericytes. Moreover, ahMNCs had much more *in vitro* differentiation potentials into neural cell lineages than pericytes and the other cell types. Compared with MSCs, ahMNCs showed little *in vitro* differentiation capacity into osteogenic and adipogenic cells. In addition, significantly higher excretion levels of MCP-1 and GRO family proangiogenic factors were observed in ahMNCs compared with those in fNSCs and MSCs. Taken together, these results indicate that ahMNCs have their own features that can differentiate ahMNCs from other cell types and NSCs from other sources [23, 24, 26, 29, 51–54].

In this study, three batches of ahMNCs (001TL, 008TL, and 015TL) were utilized. The three batches showed similar molecular and functional properties, such as cellular morphology, proangiogenic effects, expression of cell type-specific markers and differentiation potentials into neural cells. However, mRNA levels of cell type markers and mRNA and protein levels of several cytokines were significantly different. The differences might indicate variations among batches of ahMNCs, which might influence on the therapeutic effects and treatment mechanisms of ahMNCs. However, the differences were not observed consistently when they were examined by different experimental techniques. Moreover, the differences among the three batches of ahMNCs were still lesser than those among different cell types, which were even validated by other experimental techniques. These results suggest that the three batches of ahMNCs share common characteristics which might be different from those of other cell types. The differences among batches of ahMNCs might originate from various experimental factors, which need to be elucidated further.

Flow cytometry analysis and ICC results in this study demonstrated that identity of cells and their differentiation potentials should be tested using multiple experimental techniques with appropriate positive and negative controls. All cell types examined in this study commonly expressed high levels of MSC markers (such as CD29, CD44, CD73, CD90, and CD105) and Nestin, an NSC marker. The expression of Nestin as a NSC marker in MSCs and endothelial cells is controversial. The developmental origin of DPSCs is the neural crest which differentiates into neural-lineage cells [55], which could be the reason why DPSCs are positive for Nestin [56]. ADSCs are MSCs derived from pericytes resident in the perivascular region of blood vessels, which are positive for Nestin [28, 57, 58]. HUVECs are also reported to be positive for Nestin [59]. These suggest that DPSCs, ADSCs, and HUVECs may be positive for Nestin. However, discrepancies in the expression of Nestin and GFAP between the results from flow cytometry and ICC might be due to differences in the sensitivity of antibodies. Therefore, identification of a specific cell type should not be dependent on the expression of a marker or sole experimental technique.

After induction of differentiation, DPSCs, ADSCs, and HUVECs were positive for Tuj1 and MAP2, the neuron markers. However, their levels were much weaker than those in fNSCs and ahMNCs. In addition, morphologies of pericytes, endothelial cells, and MSCs after differentiation were different from those of neural cells. Previously, electrophysiological analysis of neuron-like cells differentiated from ahMNCs was conducted, which showed KCl-induced Ca^2+^ transient [15]. The results indicated that differentiation of MSCs into neurons might be incomplete. Several reports also demonstrated that Tuj1-positive neuron-like cells derived from nonneural-lineage stem cells, such as MSCs do not have an activity of functional neurons [60, 61]. In the neural differentiation condition of this study, most of HUVECs were floating to be dead and remained cells did not have typical neuron-like morphologies suggesting functional limitation since morphology and function are closely related, especially in neuron [62]. Moreover, without a specific inducer [63], the possibility of *trans*-differentiation of HUVECs into neurons is very low. The contamination by neurons during primary culture of HUVECs is also rarely possible considering the components of umbilical cord.

In this study, ahMNCs showed few *in vitro* differentiation potentials into osteogenic and adipogenic cells, which is a unique characteristic of mesenchymal-lineage cells. MSC-like properties of NSCs derived from adult human brains have been demonstrated by several previous studies [21–28, 64, 65]. Especially, Behnan et al. [64] have examined effects of *in vitro* culture conditions on features of adult human neural progenitor cells (NPCs) derived from epilepsy surgery. As we reported [15], NPCs could not be maintained in a sphere-forming culture condition while NPCs could proliferate stably in attachment culture conditions [64, 66–68]. However, attachment conditions altered gene expression of NPCs to make NPCs have mesenchymal-neuroectodermal hybrid nature [64, 65]. Although we could not find robust differentiation potentials of ahMNCs into mesenchymal-lineage cells, expression of MSC markers of ahMNCs indicated that the attachment culture condition might change *in vitro* characteristics of ahMNCs as well.

This study demonstrated that ahMNCs took part in structural formation of vessels in the location of pericytes and that proangiogenic effects of ahMNCs might be mediated by their unique paracrine factors, MCP-1 and GRO family, using *in vivo* Matrigel plug assay. Red blood cells (RBCs) in the vessels in Matrigel indicated anastomosis between new vessels in Matrigel and surrounding host vessels. The anastomosis is the most obvious proof of functionality of new vessels. Moreover, MCP-1 and GRO family might exert their proangiogenic effects since neutralizing antibodies for MCP-1 and GRO family reduced *in vivo* proangiogenic effects of ahMNCs. Compared with ahMNCs, fNSCs expressed lower levels of paracrine factors, which might be the reason why fNSCs did not have proangiogenic effects in the *in vivo* Matrigel plug assay. MCP-1, also known as CCL2, is a potent cytokine that attracts monocytes across vascular endothelial cells. It is also an angiogenic factor that can increase collateral vessel formation and blood flow to the ischemic tissue [40–44]. MCP-1 can also directly act on endothelial cells to induce angiogenesis via MCP-1-induced protein (MCPIP) [41]. Angiogenic effects of GRO family are mainly reported in tumor-associated angiogenesis. GRO family exerts its angiogenic effects via its CXCR2 receptor [44–50].

## 5. Conclusion

Taken together, results in this study demonstrated unique features of ahMNCs compared with various cell types that could be utilized in regenerative medicine. Specific properties of ahMNCs were different from those of fNSCs, strongly indicating that human NSCs derived from different sources could have their distinct characteristics which could influence therapeutic effects and treatment mechanism of NSCs. Based on expression of proangiogenic paracrine factors of ahMNCs, ahMNCs could be utilized for diseases that require angiogenesis to recover.

## Data Availability

The analyzed data sets generated during the study are available from the corresponding author on reasonable request.

## Supplementary Materials

Supplementary Materials Supplementary file includes flow cytometric analysis between ahMNCs (001TL, 008TL, and 015TL) and other control cells (fNSC, HUVEC, ADSC, DPSC, and hPC-PL). The tables show the information of antibodies, primers, and cytokine array data.
