# Supplementary material for: Adult Human Multipotent Neural Cells Could Be Distinguished from Other Cell Types by Proangiogenic Paracrine Effects via MCP-1 and GRO
Source: Stem Cells Int. 2021 Aug 12;2021:6737288. doi: 10.1155/2021/6737288 (PMC8380502; doi:10.1155/2021/6737288)
Supplement: Supplementary Materials — Supplementary file includes flow cytometric analysis between ahMNCs (001TL, 008TL, and 015TL) and other control cells (fNSC, HUVEC, ADSC, DPSC, and hPC-PL). The tables show the information of antibodies, primers, and cytokine array data. [file 6737288.f1.docx]

**
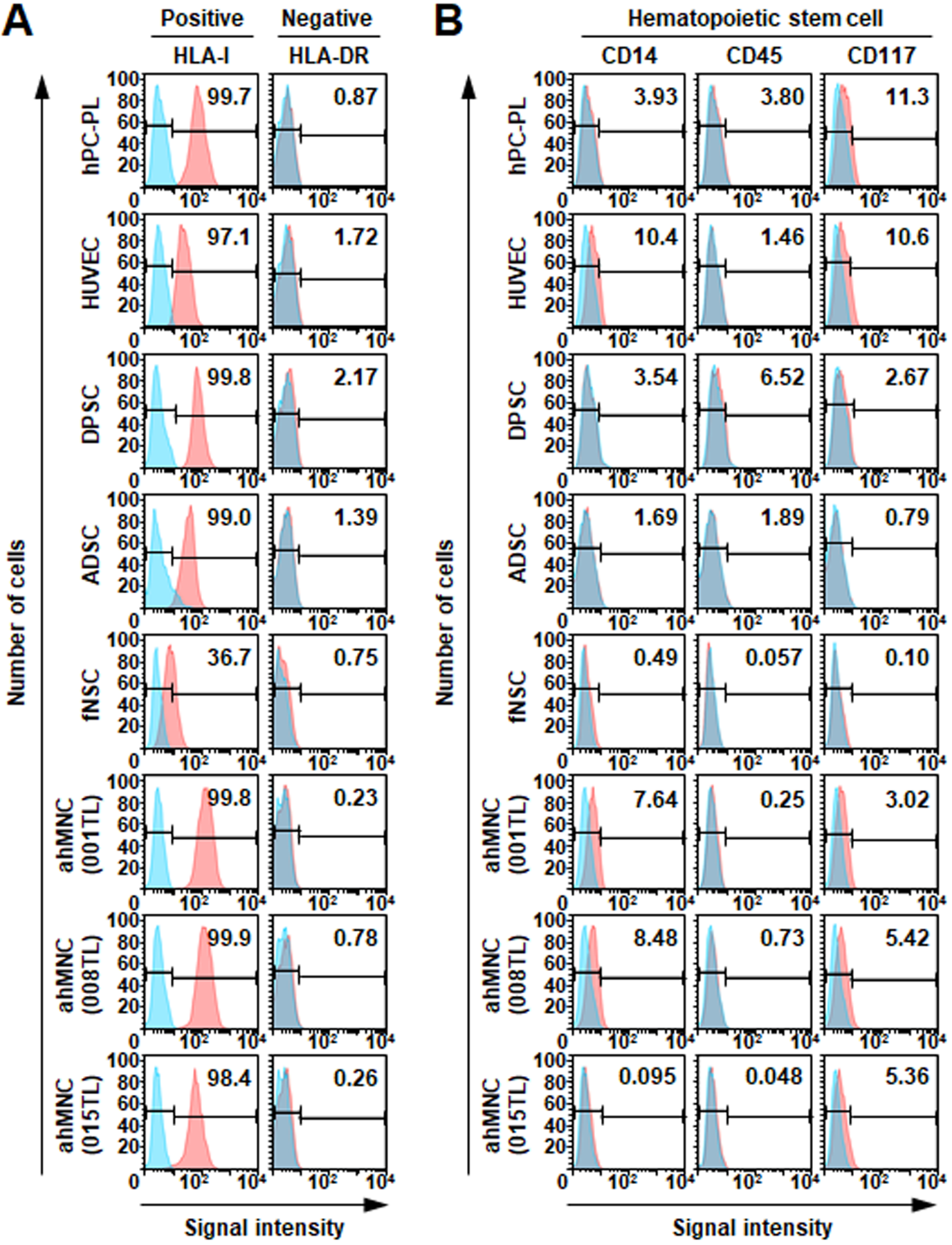
**

**Figure S1. Flow cytometric analysis of HLA-1, HLA-DR and HSC markers.** Expression levels of HLA-1 (positive control), HLA-DR (negative control) (**A**) and HSC markers (CD14, CD45, and CD117) (**B**) were analyzed by flow cytometry. Number in each panel indicates percent of marker-positive cells.

| **Table SI. Primary antibodies used for immunofluorescence and immunocytochemistry** | | | | | | | | | | | |  |
| --- | --- | --- | --- | --- | --- | --- | --- | --- | --- | --- | --- | --- |
| **Antibodies** | | **Maker** | | | | | **Cat. No.** | | **Dilution ratio** | | |  |
| GFP | | abcam | | | | | ab13970 | | 1:500 | | |  |
| CD31 / PECAM-1 | | Santa Cruz Biotechnology | | | | | sc-1506 | | 1:50 | | |  |
| αSMA | | Dako | | | | | M0851 | | 1:400 | | |  |
| Nestin | | Thermo Fischer Scientific | | | | | MA1-110 | | 1:500 | | |  |
| GFAP | | abcam | | | | | ab4674 | | 1:2000 | | |  |
| Tuj1 | | abcam | | | | | ab78078 | | 1:1000 | | |  |
| MAP2 | | abcam | | | | | ab32454 | | 1:1000 | | |  |
| GFP, green fluorescent protein; CD, cluster of differentiation; αSMA, alpha-smooth muscle actin; GFAP, glial fibrillary acidic protein; Tuj1, neuron-specific class III beta-tubulin; MAP2, microtubule-associated protein 2 | | | | | | | | | | | |  |
| **Table SII. Primer sequences for qRT-PCR** | | | | | | | | | | | | |
| **Genes** | **Size**  **(bp)** | | | **Sequences** | | | | | | | **Tm (°C)** | |
| NG2 | 277 | | | F | ACTGGCTAGGGGTGTCAATG | | | | | | 60 | |
|  |  |  |  | R | TCCTCAAGGTCCTGCTGAGT | | | | | |  |  |
| PDGFRβ / CD140b | 286 | | | F | GACAGGGAGGTGGATTCTGA | | | | | | 60 | |
|  |  |  |  | R | AGGTGTAGGTCCCCGAGTCT | | | | | |  |  |
| CD146 | 101 | | | F | ACTTCCACCTCCACCAGCTC | | | | | | 60 | |
|  |  |  |  | R | GTCTGCGCCTTCTTGCTC | | | | | |  |  |
| αSMA | 256 | | | F | CCGGGAGAAAATGACTCAAA | | | | | | 60 | |
|  |  |  |  | R | GAAGGAATAGCCACGCTCAG | | | | | |  |  |
| GAPDH | 180 | | | F | ATGGGGAAGGTGAAGGTCGG | | | | | | 60 | |
|  |  |  |  | R | GACGGTGCCATGGAATTTGC | | | | | |  |  |
| NG2, neuron-glial antigen 2; PDGFRβ, platelet derived growth factor receptor-beta; CD, cluster of differentiation; αSMA, alpha-smooth muscle actin; GAPDH, glyceraldehyde-3-phosphate dehydrogenase | | | | | | | | | | | | |
| **Table SIII. Primary antibodies used for flow cytometry** | | | | | | | | | | | |  |
| **Antibodies** | | | **Conjugation** | | | **Maker** | | **Cat. No.** | | **Dilution ratio** | |  |
| NG2 | | | Alexa Fluor 488 | | | BD Bioscience | | 562413 | | 1:100 | |  |
| PDGFRβ / CD140b | | | PE | | | BD Bioscience | | 558821 | | 1:100 | |  |
| CD146 | | | Alexa Fluor 647 | | | BD Bioscience | | 563619 | | 1:100 | |  |
| CD31 | | | FITC | | | BD Bioscience | | 558068 | | 1:100 | |  |
| CD29 | | | PE | | | BD Bioscience | | 555443 | | 1:100 | |  |
| CD44 | | | APC | | | BD Bioscience | | 559942 | | 1:100 | |  |
| CD73 | | | PE | | | BD Bioscience | | 550257 | | 1:100 | |  |
| CD90 | | | FITC | | | BD Bioscience | | 555595 | | 1:100 | |  |
| CD105 | | | APC | | | BD Bioscience | | 562408 | | 1:100 | |  |
| CD14 | | | FITC | | | BD Bioscience | | 555397 | | 1:100 | |  |
| CD45 | | | FITC | | | BD Bioscience | | 555482 | | 1:100 | |  |
| CD117 | | | PE | | | BD Bioscience | | 555714 | | 1:100 | |  |
| Nestin | | | APC | | | BD Bioscience | | 51-9007230 | | 1:500 | |  |
| GFAP | | | APC | | | BD Bioscience | | 51-9007228 | | 1:500 | |  |
| Tuj1 | | | FITC | | | BD Bioscience | | 560381 | | 1:500 | |  |
| O4 | | | PE | | | R&D Systems | | FAB1326P | | 1:100 | |  |
| HLA-I | | | FITC | | | BD Bioscience | | 555551 | | 1:100 | |  |
| HLA-DR | | | APC | | | BD Bioscience | | 559866 | | 1:100 | |  |
| NG2, neuron-glial antigen 2; PDGFRβ, platelet derived growth factor receptor-beta; CD, cluster of differentiation; GFAP, glial fibrillary acidic protein; Tuj1, neuron-specific class III beta-tubulin; HLA, human leukocyte antigen | | | | | | | | | | | |  |

| **Table SIV. Statistical comparison of cytokine levels of 001TL, 008TL, and 015TL** | | | |
| --- | --- | --- | --- |
|  | 001TL vs. 008TL | 001TL vs. 015TL | 008TL vs. 015TL |
| MCP-1 / CCL2 | 0.64 | 0.07 | 0.07 |
| IL-8 / CXCL8 | 0.01 | 0.15 | 0.80 |
| GRO α/β/γ / CXCL1/2/3 | 0.06 | 0.34 | 0.02 |
| FGF basic / bFGF / FGF-2 | 0.03 | 0.15 | 0.47 |
| IL-6 | 0.36 | 0.00 | 0.00 |
| TIMP-1 | 0.03 | 0.66 | 0.18 |
| TIMP-2 | 0.57 | 0.37 | 0.29 |
| GRO α / CXCL1 | 0.14 | 0.18 | 0.86 |
| EGF | 0.00 | 0.00 | 0.02 |
| IL-1 R4 | 0.03 | 0.00 | 0.08 |
| CTACK / CCL27 | 0.01 | 0.14 | 0.02 |
| NT-3 | 0.00 | 0.03 | 0.07 |
| OPG / Osteoprotegerin / TNFRSF11B | 0.10 | 0.00 | 0.01 |
| IGFBP-2 | 0.02 | 0.02 | 0.04 |
| OSM / Oncostatin M | 0.01 | 0.00 | 0.02 |
| IGFBP-1 | 0.01 | 0.02 | 0.04 |
| MIP-1 β / CCL4 | 0.00 | 0.00 | 0.01 |
| IGFBP-6 | 0.01 | 0.01 | 0.02 |
| Lymphotactin / XCL1 | 0.10 | 0.10 | 0.17 |
| NAP-2 / PPBP / CXCL7 | 0.00 | 0.01 | 0.03 |
| VEGF-A | 0.03 | 0.00 | 0.01 |
| IL-1 ra / IL-1 F3 | 0.01 | 0.00 | 0.00 |
| FGF-9 | 0.00 | 0.01 | 0.01 |
| VEGF-D | 0.04 | 0.00 | 0.21 |

Numbers indicates P values.
